# Conceptualizing the evolutionary quantitative genetics of phenological life‐history events: Breeding time as a plastic threshold trait

**DOI:** 10.1002/evl3.278

**Published:** 2022-04-05

**Authors:** Jane M. Reid, Paul Acker

**Affiliations:** ^1^ Centre for Biodiversity Dynamics NTNU Trondheim 7491 Norway; ^2^ School of Biological Sciences University of Aberdeen Aberdeen AB24 2TZ United Kingdom

**Keywords:** Additive genetic variance, breeding date, gene‐by‐environment interaction, phenology, phenotypic plasticity, quantitative genetics, reaction norm, threshold trait

## Abstract

Successfully predicting adaptive phenotypic responses to environmental changes, and predicting resulting population outcomes, requires that additive genetic (co)variances underlying microevolutionary and plastic responses of key traits are adequately estimated on appropriate quantitative scales. Such estimation in turn requires that focal traits, and their underlying quantitative genetic architectures, are appropriately conceptualized. Here, we highlight that directly analyzing observed phenotypes as continuously distributed quantitative traits can potentially generate biased and misleading estimates of additive genetic variances and individual‐by‐environment and gene‐by‐environment interactions, and hence of forms of plasticity and genetic constraints, if in fact the underlying biology is best conceptualized as an environmentally sensitive threshold trait. We illustrate this scenario with particular reference to the key phenological trait of seasonal breeding date, which has become a focus for quantifying joint microevolutionary, plastic, and population responses to environmental change, but has also become a focus for highlighting that predicted adaptive outcomes are not always observed. Specifically, we use simple simulations to illustrate how potentially misleading inferences on magnitudes of additive genetic variance, and forms of environmental interactions, can arise by directly analyzing observed breeding dates if the transition to breeding in fact represents a threshold trait with latent‐scale plasticity. We summarize how existing and new datasets could be (re)analyzed, potentially providing new insights into how critical microevolutionary and plastic phenological responses to environmental variation and change can arise and be constrained.

**Impact Summary**

Will wild populations persist or be driven to extinction given rapidly changing environmental conditions? We urgently need to answer this question, and identify mechanisms that facilitate population persistence.

Wild populations could in principle survive environmental changes through adaptive genetic evolution, and/or because individuals can directly modulate key characteristics (termed “traits”) that allow them to maintain high survival and reproductive success (resulting in adaptive “phenotypic plasticity”). Successfully predicting population outcomes therefore requires that we can adequately quantify forms of plasticity and underlying heritable genetic variation in key environmentally sensitive traits in wild populations. This in turn requires that we conceptualize, measure, and analyze key traits in ways that reasonably represent true underlying biology; it should otherwise be no surprise if predicted outcomes do not materialize in nature.

Here, we highlight how misleading conclusions on forms of available genetic variation and plasticity can potentially arise if traits are analyzed as directly inherited continuously distributed phenotypes when in fact the underlying biology is better represented as a plastic “threshold trait” where environmental changes can cause individuals to switch between discrete alternative states.

We highlight this situation by considering seasonal breeding date, which has become a key focal trait for understanding microevolutionary, plastic, and population responses to environmental changes, but has also become a focus for highlighting that predicted responses are commonly not fully observed. We show that the common practice of directly analyzing observed breeding dates can yield misleading inferences on available genetic variation and plasticity if individuals experience environmentally induced switches to breeding where date per se has little or no direct causal effect.

We outline how future analyses of timings of breeding and other life‐history events could be reconceptualized and reimplemented, thereby potentially providing new insights into constraints on phenological changes and unifying different observed reproductive outcomes into a single conceptual framework.

The ultimate persistence of populations experiencing changing environments and resulting maladaptation is expected to depend on forms and magnitudes of phenotypic plasticity and microevolutionary change, including microevolution of plasticity, in key environmentally sensitive traits that affect fitness (Chevin et al. 2010; Kelly 2019; Radchuk et al. 2019). Accordingly, ongoing ambitions in evolutionary ecology are to quantify additive genetic variances and components of selection affecting key traits, and affecting reaction norms that define trait plasticity across environments, in wild populations (e.g., Charmantier and Garant 2005; Husby et al. 2011; Merilä and Hendry 2014; Lande 2015; Arnold et al. 2019; Ramakers et al. 2019; de Villemereuil et al. 2020). Forms and rates of phenotypic change, and resulting population outcomes, can then in principle be predicted (Chevin et al. 2010; Gienapp et al. 2013; Radchuk et al. 2019; Simmonds et al. 2019a). Yet, despite advances in estimating key effects and predicting joint consequences, one common conclusion is that microevolutionary and phenotypic responses to apparent selection that are predicted in wild populations are not fully observed (Merilä et al. 2001; Walsh and Blows 2009; Pujol et al. 2018).

Core principles of microevolution and phenotypic plasticity in quantitative traits are well understood theoretically and have been extensively validated through structured and selective breeding in experimental and domesticated populations (Scheiner 1993, 2002; Lynch and Walsh 1998). Persistent discrepancies in wild population studies therefore imply that estimates of available additive genetic variation in and/or effective strengths of selection on focal traits or trait plasticities are biased, and/or that additional processes that constrain mean genetic or phenotypic values are ignored. Indeed, there are multiple well‐established reasons why such limitations might arise (Merilä et al. 2001; Pujol et al. 2018; Bonnet et al. 2019). Not least, biases can result from nonrandom observation failure and/or error in phenotypes, fitness, or relatedness, and from common environmental effects that confound estimated genetic effects (Kruuk and Hadfield 2007; Hadfield 2008; Wolak and Reid 2017). Gene flow, spatial and/or temporal environmental variation, and skewness in breeding values can all maintain systems at equilibria away from apparent current local optima (Lande 2015; Bonamour et al. 2017; Reid et al. 2021). Meanwhile, genetic correlations among multiple traits and/or across sexes can limit available additive genetic variation in the direction of selection and generate opposing components of indirect selection, and thereby constrain evolution (Kruuk et al. 2008; Walsh and Blows 2009).

Yet, persistent discrepancies between estimated and true quantitative genetic parameters, and hence between predicted and observed microevolutionary and phenotypic responses, could also arise if the true biological forms of focal traits differ from how those traits are typically conceptualized and analyzed. Measured axes of apparent phenotypic and genetic variation might then not map directly or linearly onto true biological scales on which evolution could actually occur. Such divergence between conceptualization and reality could directly cause biased estimates of key parameters, and potentially also generate artifactual instances of apparent missing data and skewness and hidden genetic covariances and environmental effects that could cause further discrepancies. Some reformulation of conceptual frameworks, and re‐analyses of empirical datasets, would then be required to draw appropriate inferences and predict system outcomes.

Here, we highlight how such discrepancies could potentially arise if traits that are typically conceptualized and analyzed as continuously distributed phenotypes, where genetic and environmental effects are envisaged to act directly on observed phenotypic scales, are in fact more appropriately conceptualized and analyzed as plastic threshold traits with observed switches between discrete states. Genetic and environmental effects could then act on underlying latent scales, meaning that effects that are directly estimated on observed phenotypic scales could generate biased and misleading predictions.

We illustrate this situation with specific reference to the seasonal timing of breeding. Here, breeding time is a key phenological trait that has become a major focus for conceptual and empirical developments in understanding microevolutionary and plastic responses to environmental change and resulting population outcomes (Charmantier et al. 2008; Husby et al. 2011; Gienapp et al. 2013; Bonnet et al. 2019; Radchuk et al. 2019; Simmonds et al. 2019a; Visser and Gienapp 2019). Yet, breeding time has also become a focus for highlighting that microevolutionary and phenotypic changes that are predicted in wild populations are often not observed and/or are apparently insufficient responses to environmental changes (Price et al. 1988; Merilä et al. 2001; Gienapp et al. 2006, 2013; Charmantier and Gienapp 2014; Bonamour et al. 2017).

Specifically, we use simple simulations to illustrate that treating breeding time as a continuously distributed trait that is directly expressed on the observed scale of ordinal calendar dates could generate misleading inferences on magnitudes of available additive genetic variation and forms of environment dependence if the transition from not breeding to breeding is in fact best represented as a plastic threshold trait. We outline how reconceptualized analyses could be implemented, and thereby highlight opportunities to reevaluate evolutionary potential, encompassing plasticity, while minimizing bias and unifying currently distinct concepts of different reproductive outcomes in evolutionary ecology.

## Current Quantitative Genetic Conceptualizations of Breeding Time

Seasonal timings of vegetation growth and peak invertebrate abundance are commonly advancing with climate warming, raising key questions of whether higher trophic‐level species can advance their breeding times (or other life‐history events) sufficiently to maintain temporal synchrony between peak resource demand and supply, and hence maintain population growth (Gienapp et al. 2013; Renner and Zohner 2018; Inouye et al. 2019; Simmonds et al. 2019a; Visser and Gienapp 2019; Samplonius et al. 2021). Such advances could in principle occur through combinations of adaptive microevolution and phenotypic plasticity in breeding time, including microevolution of forms of plasticity (Brommer et al. 2008; Husby et al. 2010; Gienapp et al. 2013; Charmantier and Gienapp 2014; Phillimore et al. 2016).

To examine these possibilities, leading empirical studies have used the best available individual‐based datasets to estimate key quantitative genetic parameters underlying observed variation in breeding time in wild vertebrate populations (e.g., Merilä et al. 2001; Gienapp et al. 2006; Brommer et al. 2008; Charmantier et al. 2008; Husby et al. 2010, 2011; Germain et al. 2016; Bonnet et al. 2019; Ramakers et al. 2019; Evans et al. 2020; Biquet et al. 2021). Here, breeding time is treated as a continuously distributed trait that is directly measured and analyzed on scales of ordinal dates, or days since some arbitrary seasonal start time. Raw distributions of observed breeding dates are typically taken as sufficiently close to Gaussian to employ linear mixed models to directly estimate phenotypic and additive genetic variances, including variances in linear reaction norm slopes describing labile plasticity (i.e., within‐individual phenotypic variation; Gienapp et al. 2006; Brommer et al. 2008; Charmantier et al. 2008; Husby et al. 2010; Porlier et al. 2012; Ramakers et al. 2019; but see Bonamour et al. 2017; Bonnet et al. 2019).

**Figure 1:**
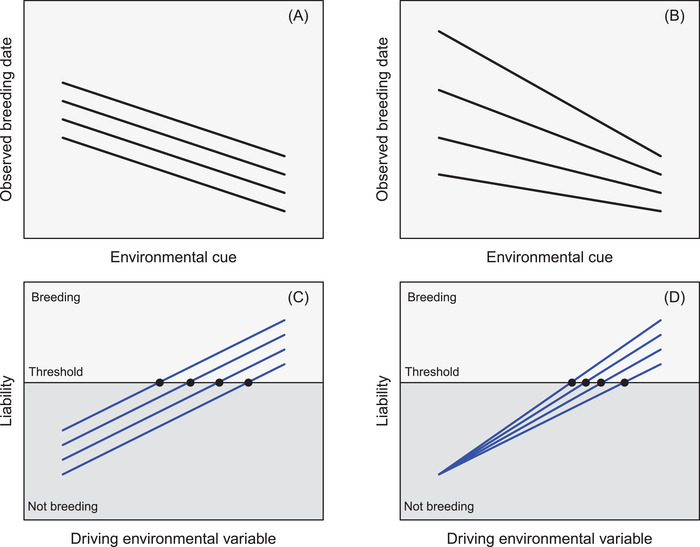
Concepts of breeding date as: (A and B) a plastic phenotypic trait that is directly expressed on the scale of ordinal dates and varies following some defined environmental “cue,” with reaction norm slope for labile plasticity that is (A) constant or (B) varies among individuals (black lines). (C and D) a plastic threshold trait where individual liability varies with some driving environmental variable, with (C) constant liability‐scale reaction norm slope but intercepts that vary among individuals, or (D) constant liability‐scale reaction norm intercept but slopes that vary among individuals (blue lines). Black points highlight each individual's transition from nonbreeding (dark gray, below the threshold) to breeding (light gray, above the threshold), which is then observed to occur on a (noncausal) date. Reaction norms could be any shape, but linear forms are shown for simplicity. Environmental “cues” are envisaged to act during discrete prebreeding time windows, whereas “driving environmental variables” are envisaged to directly affect liabilities throughout the focal seasonal period. Both could concern the same entity (e.g., temperature).

This approach, and any subsequent microevolutionary prediction, rests on a core assumption that additive genetic and environmental effects act directly on the observed date or day scale, with reaction norm slopes that directly describe changes in breeding date in relation to defined environmental “cues” (Figure 1A). Such reaction norm slopes could vary among individuals (i.e., individual‐by‐environment interactions, I×E) and potentially show additive genetic variation, implying the existence of gene‐by‐environment interactions (G×E). The magnitude of additive genetic variance in breeding date could then differ among environments, generating environment‐specific evolutionary potential (Figure 1B; Husby et al. 2010, 2011). Indeed, breeding date and other phenological traits are widely used as illustrative examples in reviews that conceptualize variation in reaction norm slopes for labile plasticity, and illustrate how to identify environmental cues acting within defined time windows that predict plastic outcomes (e.g., Nussey et al. 2007; Phillimore et al. 2013; Bonamour et al. 2019; Simmonds et al. 2019b).

Resulting analyses commonly report moderate additive genetic variance and heritability in breeding date (i.e., effectively in reaction norm elevation; Merilä et al. 2001; Gienapp et al. 2006; Bonnet et al. 2019; Biquet et al. 2021), sometimes including associative genetic effects of females’ socially paired males (Germain et al. 2016; Evans et al. 2020). There is commonly evidence of substantial among‐individual variance in reaction norm slope (i.e., I×E), with some reported evidence of additive genetic variance (i.e., G×E; Brommer et al. 2008; Husby et al. 2011; but see Charmantier et al. 2008). However, estimated effects and apparent environmental cues are not always consistent within single species, even among analyses of the same or adjacent populations (e.g., Husby et al. 2010; Porlier et al. 2012; Bonamour et al. 2019). Such differences may stem from analytical differences (Ramakers et al. 2019). Yet, recent advanced analyses of additive genetic variance and G×E, and of the magnitude and consistency of directional selection for earlier breeding, have still not fully resolved why phenological mismatches persist (e.g., Ramakers et al. 2019; de Villemereuil et al. 2020). One possibility is that discrepancies between predicted and observed microevolutionary changes in breeding date, and inconsistent estimates of G×E, could arise partly because current typical conceptualizations of breeding date as a directly expressed quantitative trait substantively differ from biological reality, meaning that existing analyses do not adequately capture true biological effects.

## Conceptualizing Breeding Date as the Manifestation of a Plastic Threshold Trait

The standard assumption that individuals have genetic values that directly and additively affect their breeding date, and hence that phenotypic and evolutionary responses directly occur on the linear observed scale of ordinal dates, could in principle be correct. However, it can be questioned whether “date” is in fact a directly evolving phenotypic trait, rather than simply a useful human measure of time. It may often be more biologically relevant to view an individual's phenotype at any seasonal timepoint as either “not breeding” or “breeding.” Individuals then switch between these phenotypic states at some point, generating an observable date on which breeding commences that emerges as a side effect rather than as a directly evolving entity. Additive genetic effects, microevolution, and plasticity could then act on the scale of the process that underlies the phenotypic state transition, not on “date” directly. Onset of breeding can then be conceptualized as the manifestation of labile plasticity in a threshold trait.

The general concept of a threshold trait is well established in evolutionary biology, and can apply to quantitative genetic traits that display approximately dichotomous alternative phenotypes (Falconer and Mackay 1996, Ch. 18; Roff 1996; Lynch and Walsh 1998, Ch. 25; Reid and Acker 2022). Briefly, individuals are envisaged to have a latent “liability” that translates into expression of alternative phenotypes (e.g., “not breeding” vs. “breeding”) when below versus above a threshold (Figure 1C). Liabilities can vary with environmental variables following reaction norms that act on the latent liability scale (i.e., “latent plasticity”) rather than on any directly observable phenotypic scale (Reid and Acker 2022). Liability‐scale reaction norm intercepts and slopes could show additive genetic and/or environmental variation and hence vary among individuals or lineages (Figure 1C,D), and potentially also show liability‐scale I×E and G×E.

Given this conceptual model of a plastic threshold trait, there is no explicit date axis in the switch from not breeding to breeding (Figure 1C,D). Rather, breeding date emerges as the time (which could be recorded on any arbitrary scale) when an individual's liability reaches a sufficient value in response to the driving environmental variable(s) (given the liability‐scale reaction norm) to cross the threshold (Figure 1C,D). Except in the specific and perhaps unlikely situation where a sole driving environmental variable exists and is perfectly linearly related to date (or is in fact date), values of additive genetic variance, I×E, and G×E estimated on the scale of observed breeding dates could be biased and misleading with respect to the true values on the latent liability scale that, in general, is the scale on which microevolution of non‐Gaussian traits is most sensibly quantitatively predicted (de Villemereuil et al. 2016).

Such outcomes can be illustrated through simple simulations that show what could be inferred by directly examining variation in observed breeding date, as is typically done, if in fact the transition to breeding behaves as a plastic threshold trait. Key points are summarized below, with full code and details of illustrative parameterizations in Supporting Information.

## Illustrating the Consequences of Alternative Trait Conceptualizations

### BASIC THRESHOLD TRAIT MODEL WITH VARIANCE IN LIABILITY‐SCALE INTERCEPT

Consider a basic scenario where the seasonal transition from not breeding to breeding behaves as a plastic threshold trait with nonzero among‐individual variance in liability‐scale reaction norm intercept α. Such variance could represent additive genetic and/or environmental effects on individuals. Meanwhile, the liability‐scale reaction norm slope β has a constant fixed value that is identical for all individuals (i.e., zero additive genetic and/or environmental variance, also implying zero proximate liability‐scale I×E and G×E; Figure 1C). There is a single driving environmental variable ɛ that is a simple linear function of, and hence perfectly correlated with, calendar date. Each individual's liability *l_i_* increases from its intercept α*_i_* as a linear function of ɛ given the fixed reaction norm slope β (Supporting Information S1). Breeding commences when *l_i_* exceeds the threshold (taken as zero by convention), yielding an observable breeding date. Hence, the only source of variation in breeding date is the specified variance in liability‐scale reaction norm intercept α (Figure 1C).

**Figure 2:**
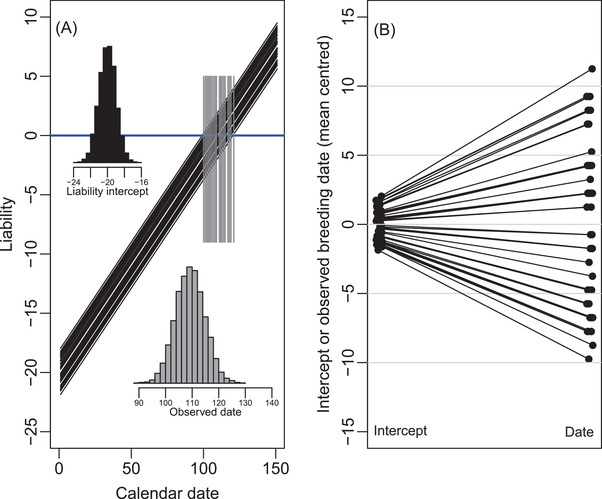
(A) Illustration of variance in observed breeding date given simulated variance in liability‐scale reaction norm intercept α but constant slope β (e.g., Figure 1C), and a driving environmental variable that is linearly related to ordinal date. Black lines show liability trajectories of 50 random lineages. Gray vertical lines highlight the points at which the liabilities cross the threshold (blue line), generating observed breeding dates. Gray and black histograms, respectively, summarize the distributions of observed breeding dates and liability intercepts across 10,000 simulated lineages. (B) Relationship between liability intercept α and observed breeding date across 50 lineages, highlighting the increase in raw variance (values are mean‐centered to facilitate comparison). For the depicted simulation, liability intercept α: realized mean (μ) −20.01; realized variance (σ^2^) 1.01; 95% CI −22.00 to −18.05; skew 0.02; mean‐standardized variance (σ^2^/μ^2^) 0.0025. Observed breeding date: mean 109.7; variance 30.1; 95% CI 99–120; skew 0.02; mean‐standardized variance 0.0025. Code and parameter values are in Supporting Information.

Simple simulations illustrate that, even given this basic scenario, the variance in observed breeding date can differ substantially from the simulated variance in α (Figure 2). This represents a rescaling, where the mean‐standardized variance in observed breeding date equals that in α (Figure 2; derivations and implications further explained in Supporting Information S2). Under these conditions (i.e., linear ɛ‐date relationship and zero variance in β), directly analyzing observed breeding date as the focal trait effectively represents an implementation of the “environmental threshold model.” Here, the trait is defined as the point on some axis of environmental variation (here taken as date) where an individual changes between dichotomous phenotypic states (e.g., Hazel et al. 2004; Tomkins and Hazel 2007; Reid and Acker 2022). However, such simple scale transformations no longer hold in more biologically realistic scenarios where ɛ is nonlinearly related to date and/or there is nonzero variance in β.

### NONLINEAR ENVIRONMENT‐DATE RELATIONSHIPS

To illustrate key points, let ɛ be a logistic function of date, broadly envisaging situations where resources available for reproduction increase rapidly at some point in spring (as commonly occurs in mid‐latitude temperate environments). The choice of a logistic function is arbitrary, but readily generates different shapes through flexible parameterizations. For example, we can consider two scenarios where the rate of increase, and hence the envisaged progression of spring, is relatively slow or fast (i.e., shallow or steep logistic slopes; Supporting Information S1). Individuals’ liabilities are initially considered to vary with ɛ through the same simple liability‐scale reaction norm as formulated above (i.e., among‐individual variation in intercept α, but constant fixed slope β). Individuals’ liability trajectories are therefore uniformly linearly related to a driving environmental variable ɛ that is itself nonlinearly related to date.

**Figure 3:**
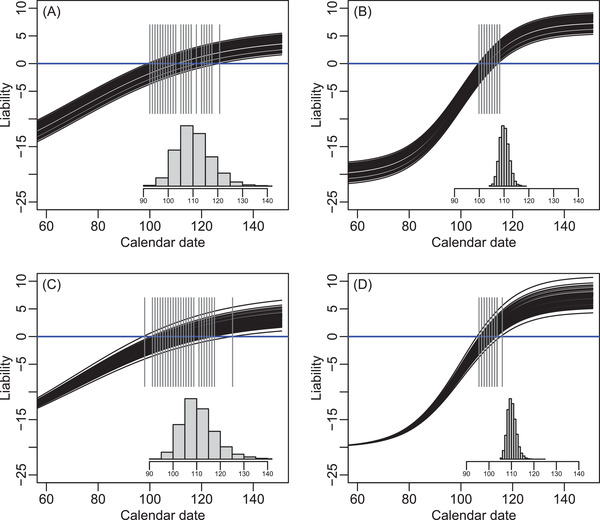
Illustrations of trajectories of liabilities and resulting variances in observed breeding date given nonzero variance in liability‐scale reaction norm (A and B) intercept α but not slope β, or (C and D) slope β but not intercept α, given driving environmental variables ɛ with (A and C) shallow or (B and D) steep logistic increases with ordinal date (Supporting Information S1). Black lines show liability trajectories of 50 random lineages. Gray vertical lines highlight the points at which the liabilities cross the threshold (blue line), generating observed breeding dates. Gray histograms summarize the distributions of observed breeding dates across 10,000 lineages. *X*‐axes are cut at day 60 to facilitate visualization. Descriptive statistics of observed breeding date for each panel: (A) mean 111.0; variance 48.9; 95% CI 99–126; skew 0.64; mean‐standardized variance 0.0040; (B) mean 110.6; variance 3.7; 95% CI 107–115; skew 0.30; mean‐standardized variance 0.0003; (C) mean 111.3; variance 53.8; 95% CI 100–128; skew 1.02; mean‐standardized variance 0.0043; (D) mean 110.7; variance 4.0; 95% CI 107–115; skew 0.62; mean‐standardized variance 0.0003. Mean‐standardized variances for A versus B and C versus D therefore differ by an order of magnitude. Realized values for simulated liability‐scale reaction norm intercepts are as in Figure 2, and for simulated slopes are mean 0.025; variance 1.57 × 10^−6^; 95% CI 0.023–0.027; skew 0.01; mean‐standardized variance 0.0025. Code and parameter values are in Supporting Information.

Resulting variances in observed breeding dates differ substantially between the two logistic scenarios (Figure 3), and also differ from the variance arising when ɛ increases linearly with date (Figure 2). This is true even though the specified means and variances in liability‐scale reaction norm parameters (α and β) are identical in all three cases. Variances in observed breeding dates can consequently be substantially larger or smaller than the simulated variance in α. For the two logistic scenarios, these differences are not simple mean rescalings (Figure 3); indeed the illustrative scenarios were designed so that resulting mean breeding dates scarcely differ (Supporting Information S1). Rather, the different variances result from the different slopes and curvatures of the relationships between ɛ and date around the zone where individuals’ liabilities approach the threshold (Figure 3). Notably, both logistic scenarios also generate right‐skewed distributions of observed breeding dates (Figure 3), even though the simulated distribution of α is Gaussian (i.e., zero skew; Figure 2; Supporting Information S1). Following the same principles, distributions of breeding dates can become further complicated and irregular, and even bimodal, if ɛ‐date relationships are irregular, which could be common in nature (Supporting Information S3).

**Figure 4:**
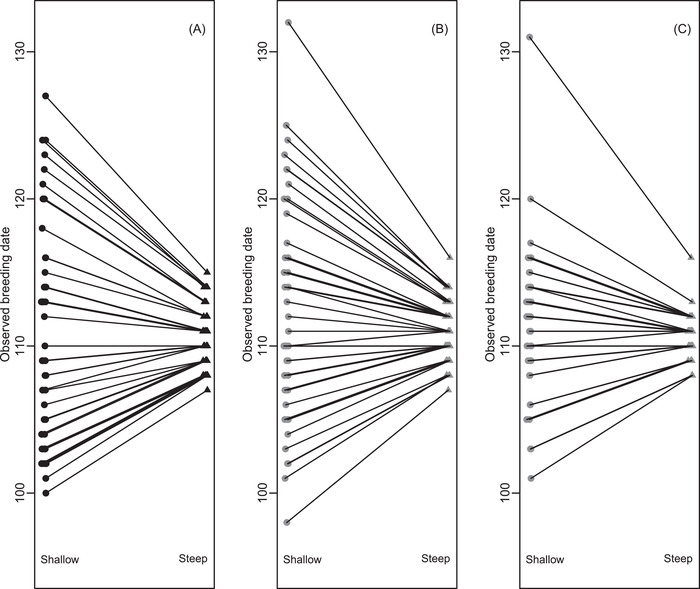
Illustrations of variances in observed breeding dates across two scenarios where the driving environmental variable ɛ is a shallow (points) or steep (triangles) logistic function of date given simulated variances in liability‐scale reaction norm (A) intercept α but not slope β, (B) slope β but not intercept α, and (C) both intercept α and slope β with negative covariance. Lines link phenotypes produced by the same individual or lineage across environmental scenarios, illustrated for 50 random lineages. Phenotypes, which could be taken to reflect underlying genetic values, are strongly correlated across environments. However, very different variances arise given shallow or steep logistic scenarios (shown by the spreads of points). These differences are similar in panels A and B, and hence irrespective of whether there is variance in α or β. Further, the difference in variance is smaller in panel C than B even though the same variance in β was simulated in both, due to the negative α‐β covariance. Variances in observed breeding dates across all 10,000 simulated lineages given shallow and steep logistic slopes are (A) 48.9 and 3.7; (B) 53.8 and 4.0; and (C) 18.5 and 1.5.

Such patterns imply that variation in the shape of the ɛ‐date relationship (i.e., in the trajectory of seasonal environmental progression) among years or locations could cause the variances in breeding dates among individuals or lineages to differ across those years or locations, even without any variation in reaction norm slope. For example, take the two illustrative logistic scenarios to represent two consecutive years, where focal individuals breed in both years. Observed breeding dates are highly correlated across simulated individuals across the 2 years (assuming individuals have constant values of α), but overall phenotypic variances differ substantially (Figure 4A).

If breeding date were conceptualized and analyzed as a continuously distributed trait that is directly expressed on the observed date scale, such patterns of environmentally induced variation in phenotypic variance would be interpreted as evidence of individual and/or genetic variation in reaction norm slope (i.e., I×E or G×E; Figure 1B). This would in turn imply opportunities for selection on and microevolution of labile phenotypic plasticity through changing reaction norm slopes (e.g., Nussey et al. 2007; Husby et al. 2010; Arnold et al. 2019; Ramakers et al. 2019). However, in fact the simulations underlying Figures 3 and 4 do not contain any variation in reaction norm slope, or hence any immediate potential for slope evolution. Rather, the apparent I×E or G×E on the observed date scale (Figure 4A) results from the interaction between variation in liability‐scale reaction norm intercept α and the changing shape of the relationship between the driving environmental variable ɛ and the biologically arbitrary scale of date (Figure 3).

Apparent evidence of conceptually interesting forms of I×E or G×E could then arise across years if the shape of the ɛ‐date relationship covaries with the mean date at which a responding liability would exceed the threshold and hence induce breeding. For example, there could be more (or less) apparent variance in breeding date in late (i.e., colder) versus early (i.e., warmer) springs, which could be taken to imply systematic environment dependence in additive genetic variance and/or opportunity for selection, and hence environment‐dependent microevolutionary potential (e.g., Charmantier and Garant 2005; Husby et al. 2011; Rowiński and Rogell 2017). This could in principle occur if, for example, early and late springs typically show steeper and shallower trajectories of ɛ, respectively (or vice versa).

**Figure 5:**
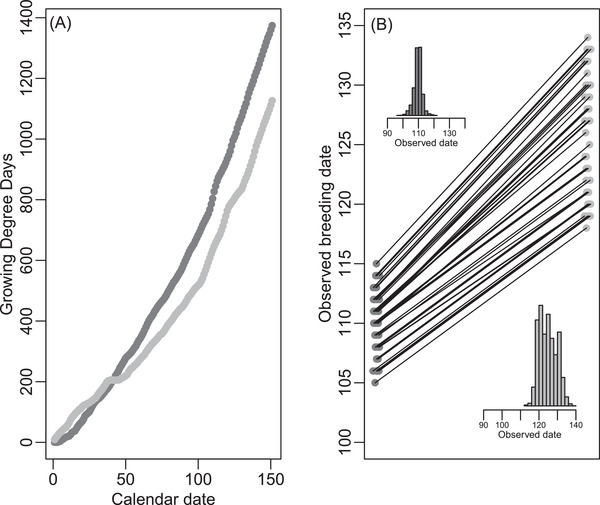
Illustrations of trajectories of (A) driving environmental variable ɛ with ordinal date in two different years, and (B) resulting liabilities and variances in observed breeding date given variance in liability‐scale reaction norm intercept α but not slope β. Dark gray and light gray points denote trajectories observed in relatively early and late springs, respectively, taken as “growing degree days” in coastal British Columbia, Canada, in 2016 and 2021. Other specifications for panel B are as in Figure 3. Mean and variance in observed breeding date are greater in the later spring, even though liability‐scale reaction norms were identical in both years with zero variance in liability‐scale reaction norm slope β. Earlier spring: mean 110.1; variance 6.6; 95% CI 104–115; skew −0.33; mean‐standardized variance 0.0005. Later spring: mean 125.3; variance 21.9; 95% CI 118–134; skew 0.26; mean‐standardized variance 0.0014.

These possibilities can be further illustrated by repeating the above simulations taking the ɛ‐date relationships as realistic seasonal trajectories of “growing degree days” (i.e., cumulative temperature) in a relatively early versus late spring (Figure 5A). Here, twice as much variance in observed breeding date emerges in the late versus early spring (Figure 5B), even though the simulations contain zero variance in liability‐scale reaction norm slope. Analogously, populations inhabiting different locations that typically experience different (variation in) trajectories of seasonal environmental progression could be inferred to have different quantitative genetic architectures for breeding time, when in fact they do not.

### (CO)VARIANCE IN LIABILITY‐SCALE INTERCEPT AND SLOPE

Very similar patterns of phenotypic variation in observed breeding date can be readily generated by repeating the above simulations with alternative parameterizations comprising nonzero variance in liability‐scale reaction norm slope β rather than in intercept α. Indeed, broad patterns are effectively indistinguishable when given nonzero variance in β but not α, as when given nonzero variance in α but not β (Figure 3A,B vs. 3C,D; Figure 4A vs. 4B). This is true even though the presence of variation in reaction norm slope versus intercept (when directly acting on observed scales) would typically be envisaged to induce very different changes in phenotypic variance across environments (e.g., Figure 1A,B).

These simple simulations also indicate that substantial variance in observed breeding date can potentially arise even with small simulated variance in β, or even in α (Figures 3, 4, 5). This is because underlying variances in reaction‐norm parameters can be substantially magnified by the shape of the ɛ‐date relationship. Hence, if there is at least some environmental variance in β or α, then additive genetic variance could be very small. Unrealistically large variation in breeding date would otherwise emerge, for example, spanning months rather than days or weeks, depending on the time course over which the liability‐scale reaction norm acts (Figure 3; Supporting Information S4).

Of course, there could be nonzero variance in both α and β and potentially also nonzero covariance, generating a nondiagonal G‐matrix for the liability‐scale reaction norm (Supporting Information S4). As with any G‐matrix, the magnitude of available additive genetic variance in any particular direction of selection could then be constrained (e.g., Walsh and Blows 2009). Reconceptualizing observed breeding date as the outcome of a plastic threshold trait with liability‐scale quantitative genetic architecture could therefore reveal how microevolutionary responses of mean breeding date could be constrained in latent multivariate space, even in the absence of any other unmeasured genetically correlated traits or among‐year environmental variation. Further, the presence of negative covariance between α and β could potentially obscure phenotypic‐scale evidence of variance in β, by the decreasing cross‐environment variation in the variance in observed breeding dates (Figure 4).

## Discussion

### IMPLICATIONS OF TRAIT CONCEPTUALIZATIONS

All measured phenotypic traits, and all quantitative genetic models used to estimate additive genetic variances and G×E interactions and predict system outcomes, are necessarily highly simplified approximations of complex biological realities (Nussey et al. 2007; Gienapp et al. 2013; Morrissey and Liefting 2016). Yet, useful predictions of microevolutionary changes in trait means and phenotypic plasticities require that models do adequately capture key aspects of system biology on appropriate quantitative scales. It should otherwise be no surprise if predicted outcomes do not materialize in nature. We highlight that the common practice of analyzing timings of life‐history events, such as seasonal commencement of breeding, as continuously distributed traits where microevolution and plasticity are envisaged to act directly on the scale of ordinal calendar dates could generate misleading inferences on apparent additive genetic variances and environmental interactions, and hence on evolutionary potentials, if such events are in fact best approximated as manifestations of environmentally sensitive threshold traits.

Specifically, our simple simulations highlight how direct analyses of observed breeding dates could yield substantially biased inferences on variances and skewness relative to underlying distributions of liability‐scale parameters, and furthermore could fail to distinguish variation in liability‐scale reaction norm intercepts versus slopes, or to identify latent multivariate constraints. Indeed, apparent evidence of I×E or G×E in phenotypic breeding date could emerge even with zero variance in liability‐scale reaction norm slope. Such outcomes arise because effects of liability‐scale reaction norms are shaped by driving environmental variables that vary nonlinearly with date, and such environment‐date relationships could vary among years or locations, whereas date per se has no causal effect on the transition to breeding.

Our current simulations are designed to illustrate basic conceptual points, not to capture likely biological effect sizes or details, or to consider further biases or challenges that could result from analyses of complex wild population datasets. Yet, the core principle that latent liability for breeding varies as some function of driving environmental variable(s), which themselves vary nonlinearly with calendar date, could commonly apply. Indeed, reproductive outcomes are widely treated as longitudinal threshold traits in animal breeding (e.g., Averill et al. 2006; Buaban et al. 2016), providing proof of concept that has scarcely been transferred into evolutionary ecology. Further, seasonal trajectories of environmental variables that affect or represent resource abundance, such as “growing degree days” and invertebrate development and abundance, are clearly nonlinearly related to date, with relationships that vary in form and timing among years and locations (e.g., Charmantier et al. 2008; Phillimore et al. 2013; Cayton et al. 2015; Shutt et al. 2019). Such nonlinear relationships could also generate misleading inferences on temperature sensitivities of phenological change (as illustrated for leaf‐out; Wolkovich et al. 2021). Although some potential driving variables could be approximately linearly related to date during key seasons (most obviously photoperiod, e.g., Gienapp et al. 2010; Phillimore et al. 2016), the fact that mean breeding date commonly varies substantially among years and adjacent habitats (e.g., Porlier et al. 2012; Bonamour et al. 2019; de Villemereuil et al. 2020) implies that effective date dependence is typically secondary to environment dependence (see also Cayton et al. 2015). Frameworks that conceptualize and predict outcomes directly on the scale of dates can then be intrinsically limited.

In such circumstances, reconceptualizing the transition to breeding as a plastic threshold trait could illuminate existing results. For example, it could straightforwardly explain how the form and magnitude of apparent I×E and G×E in breeding date can differ between (sub)populations of the same species (e.g., Husby et al. 2010; Porlier et al. 2012; Ramakers et al. 2019), even with negligible genetic differentiation. This simply requires that the focal (sub)populations experience slightly different forms of among‐year variation in seasonal environmental trajectories (e.g., due to slightly differing climates and/or habitats). It could also explain why distributions of breeding date are commonly skewed, which could itself affect microevolutionary predictions (Bonamour et al. 2017). Although there are multiple genetic and ecological reasons why skew might arise (Bonamour et al. 2017; Reid et al. 2021), our simulations highlight how skewed distributions of observed breeding dates can intrinsically arise from a threshold trait, even if underlying distributions of liability‐scale genetic and/or environmental effects are strictly Gaussian (i.e., zero skew).

### OPPORTUNITIES FOR NEW ANALYSES

Obvious advantages of treating breeding date as a continuously distributed trait with direct additive effects on the observed date scale are that microevolutionary parameters can be estimated, and responses predicted, using foundational principles of evolutionary quantitative genetics enacted through linear mixed models and associated theory (e.g., Nussey et al. 2007; Arnold et al. 2019; Radchuk et al. 2019). Indeed, such efforts have provided leading examples of how to dissect and predict microevolutionary, phenotypic, and population responses to climate change in wild populations (Charmantier et al. 2008; Husby et al. 2010; Gienapp et al. 2013; Ramakers et al. 2019). But, if this standard conceptualization in fact diverges sufficiently from biological reality to yield misleading inferences and predictions, then we should shift to conceptual and analytical frameworks designed for plastic threshold traits.

Here, individual phenotypes would be recorded as “not breeding” or “breeding” across sequences of observation occasions, alongside values of relevant environmental variables that are known or hypothesized to drive transitions to breeding. In principle, generalized linear mixed models can then be used to estimate additive genetic and environmental (co)variances in liability‐scale reaction norm intercept and slope parameters, given sufficient phenotypic observations of breeding state and environmental variables across occasions and relatives (Supporting Information S4). “Breeding date” (i.e., date of first laying or parturition, or other traits such as leaf‐out, bud‐burst, or flowering) would not explicitly appear in such analyses, and in fact would not necessarily even need to be explicitly recorded (Supporting Information S4).

Although there are well‐established formulae for transforming between phenotypic‐scale and liability‐scale heritabilities for threshold traits (Dempster and Lerner 1950; de Villemereuil et al. 2016), such transformations concern heritability of expression of the alternative dichotomous phenotypes. Scale dependence then arises because effects that are straightforwardly additive on the latent liability scale become intrinsically nonadditive on the observed phenotypic scale. However, such scale‐transformation formulae for heritability do not directly apply to the point on some environmental or date axis at which phenotype switching occurs. Direct post hoc transformations of existing (often univariate) date‐scale estimates of quantitative genetic effects on breeding date into underlying (potentially multivariate) liability‐scale reaction norm parameters are consequently unlikely to be possible. Indeed, beyond the effects illustrated by our current simple simulations, alternative trait conceptualizations could induce further complex biases in estimation of key quantities from multigenerational wild population data. Reanalyses will therefore be necessary.

As for any examination of plasticity, such analyses will require measurements of key environmental variables that are known or hypothesized to drive trajectories of liability (and resulting transitions to breeding) across appropriate time periods. Indeed, while analyses that simply estimate additive genetic variance in observed breeding date can be accomplished without explicitly considering any form of plasticity or hence environmental covariates (e.g., Germain et al. 2016; Evans et al. 2020; Biquet et al. 2021), analyses of environmentally driven responses of threshold traits cannot (Supporting Information S4). However, in contrast to current common practice, there is no need to identify discrete critical time windows within which values of particular environmental variables are postulated to act as “cues” (Figure 1; e.g., Brommer et al. 2008; Husby et al. 2010; Porlier et al. 2012; Phillimore et al. 2016; Bonamour et al. 2019; Simmonds et al. 2019b). Rather, full seasonal trajectories of such environmental variable(s) are postulated to drive full seasonal trajectories of latent liabilities (e.g., Figure 3; Supporting Information S1 and S4). This eliminates previously highlighted challenges of interpretation and projection that result from defining fixed cue time windows, which may themselves change over time (Gienapp et al. 2005, 2010, 2013; Phillimore et al. 2013; Keenan et al. 2020).

Conceptualizing and analyzing the transition to breeding as a plastic threshold trait also means that data from individuals that never breed within a focal season (and hence have no observable breeding date), or that start breeding then stop then potentially start again (e.g., early clutch abandonment followed by relaying, or increased interlaying intervals), or whose exact breeding dates were unobserved, can all be included (Supporting Information S4). Nonbreeding and unobserved individuals are typically excluded from direct analyses of breeding date, potentially biasing estimates of population‐wide genetic variance and selection. The conceptual shift to a threshold trait formulation could therefore unify breeding time, breeding cessation, repeat breeding, and nonbreeding as manifestations of effectively the same (latent) trait. All such reproductive outcomes can then emerge from a single model, potentially revealing further constraints on or drivers of microevolutionary dynamics.

### SELECTION AND EVOLUTIONARY RESPONSE

The suggestion that components of genetic and environmental variance underlying observed breeding dates (and other phenological traits) may be more appropriately estimated on latent liability scales than on observed date scales does not necessarily invalidate existing estimates of selection on breeding date. Indeed, one robust observation across diverse systems is that there is commonly directional selection for earlier breeding, where individuals that breed early within particular seasons on average produce more (surviving) offspring (e.g., Husby et al. 2011; Bonnet et al. 2019; Radchuk et al. 2019; Biquet et al. 2021). Recent advanced analyses also show that fitness commonly decreases with increasing time from estimated seasonal optima, leading to renewed conclusions that some further constraint must prevent breeding date from shifting toward the apparent optimum (de Villemereuil et al. 2020).

Formulating the transition to breeding as a plastic threshold trait reveals the possibility that multivariate constraints on available additive genetic variance in the direction of phenotypic selection could arise from the intercept‐slope G‐matrix defining the latent liability‐scale reaction norm. Future analyses should consequently aim to estimate responses to selection through multivariate latent‐scale genetic covariation, which is the scale on which quantitative genetic predictions for microevolutionary change in non‐Gaussian traits most directly apply (de Villemereuil et al. 2016). This could in principle be achieved by estimating genetic covariances between latent liability‐scale parameters underlying breeding time and expected fitness (e.g., using the Robertson‐Price covariance; de Villemereuil et al. 2016; Supporting Information S4). This effectively requires shifting advocated multivariate random regression approaches for quantifying phenotypic plasticity in phenological traits and associated selection (e.g., Nussey et al. 2007; Morrissey and Liefting 2016; Arnold et al. 2019) from the observed date scale onto the underlying latent scale. Yet, although microevolutionary changes in key parameters could then in principle be correctly predicted, resulting changes in observed breeding dates could still be idiosyncratic and not fully predictable. This is because such changes will still depend on changing shapes of ɛ‐date relationships, which themselves are not easily predictable.

### CHALLENGES

If it is indeed appropriate to reconceptualize breeding time as the manifestation of a plastic threshold trait, then new analyses that estimate liability‐scale parameters should provide new insights, but will also inevitably present challenges that necessitate careful progress.

First, methods for specifying and fitting appropriate models need to be tested. Some relevant statistical methods have already been developed and evaluated in animal breeding, for example, using longitudinal threshold models with different forms of nonlinear random regression to estimate additive genetic variance in temporal manifestation of conception or mastitis (e.g., Averill et al. 2006; Negussie et al. 2012; Buaban et al. 2016). However, there will likely be challenges of translation to wild population datasets, which typically contain many fewer individuals and observations with sparser pedigree structures and considerably more uncontrolled environmental variation. There may be ways to optimize performance by appropriately structuring and interpolating available data, potentially including multiple thresholds representing sequential stages of reproduction (Supporting Information S4). Explicit examinations of such methods, and resulting parameter identifiability, bias, and precision, are now required to establish accessible methods and guidelines for best practice.

Second, key driving environmental variables need to be identified and measured throughout focal periods of seasonal progression. Of course, liability‐scale reaction norms could comprise higher order or nonlinear functions of these variables, with complex immediate and/or lagged effects, and/or could be highly multivariate (e.g., Scheiner 1993; Gienapp et al. 2010; Westneat et al. 2019). Yet, because latent liability‐scale reaction norms are by definition not directly observable, patterns of phenotypic variation cannot be directly inspected to inform plausible models. Detailed knowledge of system ecology and reproductive biology will therefore be essential to formulate sets of appropriate models that contain appropriate environmental variables, which could then be statistically compared.

Overall, the challenge of switching from well‐established direct analyses of observed calendar dates to liability‐scale analyses of longitudinal threshold traits may seem daunting. It will certainly increase complexity of modeling, interpretation, and prediction. Yet, such reformulations also have potential to make more efficient and appropriate use of all available seasonal data, rather than the current typical focus on a single annual datapoint per breeding individual (e.g., observed breeding date). Given appropriate data structures, parameters defining reaction norms for labile plasticity could potentially be estimated within years rather than solely across years, reducing the multiyear timeframe required to draw inference. Hence, the imperative now is for the interested research community to harness collective expertise to develop, implement, and evaluate new analyses across available datasets. Direct analyses of dates can still be appropriate to answer some questions. Yet, careful (re)analyses of transitions to breeding, and other phenological traits, as plastic threshold traits could provide new insights into plastic, microevolutionary, and population dynamic responses to our rapidly changing world.

## CONFLICT OF INTEREST

The authors declare no conflict of interest.

## AUTHOR CONTRIBUTIONS

JMR conceived the ideas, undertook the simulations, and wrote the manuscript, with conceptual and editing input from PA.

## DATA ARCHIVING

There are no primary data associated with this concept manuscript. Simulation code is provided as Supporting Information.

## Supporting information

Appendix S1: Liability formulations
Appendix S2: Simple scale transformation
Appendix S3: Additional illustration
Appendix S4: Analysing the transition to breeding as a plastic threshold trait
