# Supplementary material for: Conceptualizing the evolutionary quantitative genetics of phenological life‐history events: Breeding time as a plastic threshold trait
Source: Evol Lett. 2022 Apr 5;6(3):220–33. doi: 10.1002/evl3.278 (PMC9233176; doi:10.1002/evl3.278)
Supplement: Supplementary file 1 — Appendix S1: Liability formulations Appendix S2: Simple scale transformation Appendix S3: Additional illustration Appendix S4: Analysing the transition to breeding as a plastic threshold trait [file EVL3-6-220-s001.docx]

**Supporting Information**

**Conceptualising the evolutionary quantitative genetics of phenological life-history events: breeding time as a plastic threshold trait**

Jane M. Reid & Paul Acker

**Appendix S1: Liability formulations**

For current illustrative purposes, individual i’s liability for breeding on any particular date d (l_i,d_) is given by Equation 1. Here, liability l_i,d_ is envisaged to increase from the individual’s liability-scale reaction norm intercept α_i_ following the trajectory of the driving environmental variable ɛ (which has a value ɛ_d_ on day d), given a relationship defined by the individual’s liability-scale reaction norm slope β_i_. Each individual’s transition from the phenotypic state y_i,d_ of not breeding to breeding occurs when l_i,d_ equals or exceeds the defined threshold value T. This gives the individual’s observed breeding date z_i_. Formally:

l_i,d_ = α_i_ + β_i_.ɛ_d_ Equation 1

if l_i,d_ < T, y_i,d_ = not breeding

if l_i,d_ ≥ T, y_i,d_ = breeding

Hence, observed first seasonal breeding date z_i_ is the first date on which l_i,d_ ≥ T.

The objective is to evaluate relationships between variances in observed breeding dates (z_i_) and variances (and covariances) in liability-scale reaction norm intercepts and slopes (α_i_ and β_i_). This can be readily achieved analytically in some simple scenarios, most obviously given a linear ɛ-date relationship and constant β (Supporting Information S2). However, setting up general simulations allows rapid evaluation of the implications of any form of non-linear or irregular ɛ-date relationship alongside variation in β, such as could be commonplace in nature.

R code for current simulations is provided as additional Supporting Information, with parameter specifications and values summarised in Table S1. Here, the numerical values are arbitrary. However, for current illustrative purposes, values were set to generate biologically sensible ranges of observed breeding dates given an observed ɛ-date relationship of ‘growing degree days’ (GDD), envisaging a northern hemisphere bird (as has been one primary focus for wild population quantitative genetic analyses of breeding date, including plasticity). GDD can be taken as one broad measure of the progression of spring (Figure S1). The parameterisations generate breeding dates with an approximate mean of ordinal day 110 (i.e. approximately April 20^th^). Note that the choice of logistic functions to generate current illustrative trajectories of ɛ is arbitrary; any form of ɛ-date relationship could be considered.

The code generates values for each individual’s liability-scale reaction norm intercept α and slope β, which could be envisaged as additive genetic (i.e. breeding) values, or permanent or annual environmental effects, or some combination of the two. It would be easy to explicitly simulate separate genetic and environmental components, and concepts could also be extended to consider liability-scale GxE. However, these extensions are not necessary to illustrate basic conceptual points. We consider that both α and β are normally distributed, with means and variances set so that their mean-standardised variances (i.e. σ^2^/μ^2^, giving mean-standardised ‘evolvabilities’ if simulated variance is interpreted as entirely additive genetic) are identical (Table S1). Numerous other parameterisations could be considered to generate realistic (or unrealistic) distributions of observed breeding dates, including greater variance in liability-scale reaction norm slopes β. The current code solely records the date of each individual’s first transition to breeding within a focal year. It could readily be extended to record instances of starting, stopping and restarting breeding activity, such as could arise in response to non-monotonic ɛ-date relationships (see Supporting Information S3).

**Figure S1.** Illustration of driving environmental variables (ɛ) used to illustrate consequences of treating breeding date as a directly observed continuously distributed trait versus a plastic threshold trait. Here, ɛ can be linearly related to calendar date (black) with some slope δ, or can have shallow or steep logistic relationships with date (dark and light grey). Numerical values are set to match an observed trajectory of growing degree days (and hence progression of spring, open symbols) giving a mean breeding date of day 110 (vertical dotted line) which is similar for all forms of ɛ. The dashed black line shows a 1:1 relationship between ɛ and calendar date (i.e. δ=1). Details of parameter values are in Table S1.


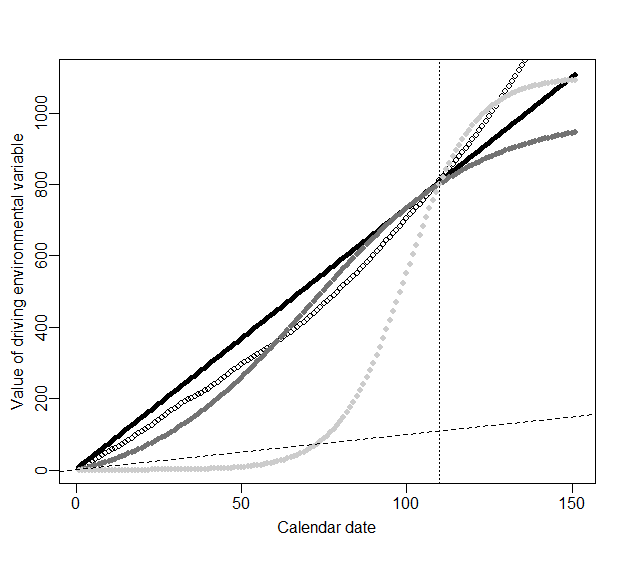


**Table S1.** Summaries of (A) equations and parameter values for relationships between driving environmental variables ɛ and ordinal date d, and (B) parameter values for liability-scale reaction norm intercept α and slope β. Both α and β are normally distributed. Values are set so that mean-standardised variances are identical (σ_α_^2^/μ_α_^2^ = 1/(-20)^2^ = 0.0025; σ_β_^2^/μ_β_^2^ = 0.00000156/0.025^2^ = 0.0025).

| **(A) Parameterisations of ɛ-date relationships** | | |
| --- | --- | --- |
| **Form** | **Equation or parameter** | **Parameter values** |
| Linear | ɛ = δ.d | gradient δ = 7.325 |
| Shallow logistic | ɛ = v/(1+e^-r(d-u)^)+y | gradient r = 0.039  midpoint u = 70  maximum v = 1055  elevation factor y = -65 |
| Steep logistic | ɛ = v/(1+e^-r(d-u)^)+y | gradient r = 0.098  midpoint u = 99  maximum v = 1100  elevation factor y = 0 |
| **(B) Parameterisations of liability-scale reaction norms** | | |
| Threshold T | Fixed value | 0 |
| Intercept α | Mean μ_α_ | -20 |
| Intercept α | Variance σ_α_^2^ | 1 |
| Slope β | Mean μ_β_ | 0.025 |
| Slope β | Variance σ_β_^2^ | 0.00000156 |
| Intercept and slope | Covariance σ_α,β_ | -0.001 |

**Appendix S2: Simple scale transformation**

Given a linear ɛ-date relationship, each individual’s observed breeding date z_i_ occurs (in fully continuous time) when its liability l_i_ equals the threshold (taken as zero), hence:

0 = α_i_ + β_i_.δ.z_i_

where δ is the slope of the linear relationship between ɛ and date (Table S1). This follows from Equation 1, since δ.z_i_ gives the value of ɛ_d_.

Hence,

z_i_ = -α_i_/ β_i_.δ

In the special case where β is a constant (i.e. zero among-individual variance in liability-scale reaction norm slope), then:

z_i_ = -α_i_/m Equation 1

where m is β.δ, which is a constant.

Then, following standard rules for variances of functions of random variables,

σ_z_^2^ = σ_α_^2^/m^2^  Equation 2

Equation 2 shows that the variance in observed breeding date (σ_z_^2^) can be substantially smaller or larger than the underlying variance in liability-scale intercept (σ_α_^2^) depending on the value of m (i.e. the overall fixed slope of the relationship between date and liability). σ_z_^2^ = σ_α_^2^ only in the special case where m=1, such that liability effectively equals date.

Further, given these same conditions,

μ_z_ = -μ_α_/m Equation 3

where μ_z_ and μ_α_ are the means (expectations) of z and α respectively.

Hence, substituting equation 3 into equation 2 for m gives

σ_z_^2^/μ_z_^2^ = σ_α_^2^/μ_α_^2^  Equation 4

Equation 4 shows that the mean-standardized variance in observed breeding date z equals the mean-standardized variance in liability-scale reaction norm intercept α.

However, this simple scale transformation only holds given the special conditions of a linear ɛ-date relationship and a constant linear reaction norm slope β. It also implicitly assumes that breeding date is measured from an appropriate origin. This is true for the current simulations, but may not be true in wild population datasets where the quantitative scale on which dates are measured (and hence the implied origin) is often arbitrary.

**Appendix S3: Additional illustration**

If there is an irregular relationship between the driving environmental variable ɛ and calendar date a bimodal distribution of observed breeding dates could potentially arise, even if the underlying distribution of liability-scale reaction norm intercepts is strictly Gaussian (e.g. Figure S2).

Such irregular ɛ-date relationships could commonly arise in nature, for example if favourable early spring conditions are followed by a cold spell that retards progress. Further, if the driving environmental variable ɛ is taken as a measure of resource availability or current environment (e.g. invertebrate abundance or current temperature) rather than a monotonically accumulating variable (e.g. growing degree days), then the ɛ-date relationship could potentially go temporarily negative (i.e. be non-monotonic). Liabilities of individuals that have already crossed the threshold could then potentially cross back again. Individuals’ reproductive activities could then start, then cease, then subsequently start again, all described by a single threshold trait model.

**Figure S2.** Illustration of trajectories of liabilities and resulting variance in observed breeding date given simulated variance in liability-scale reaction norm intercept α but not slope β, and a driving environmental variables ɛ that varies irregularly with calendar date. Black lines show liability trajectories of 50 random lineages. Grey vertical lines highlight the points at which the liabilities cross the threshold (blue line), generating observed breeding dates. The grey histogram summarises the distribution of observed breeding dates across 10000 lineages. Descriptive statistics of observed breeding date are mean 111.9; variance 45.7; 95%CI 101-123; skew 0.14; mean-standardised variance 0.0036.


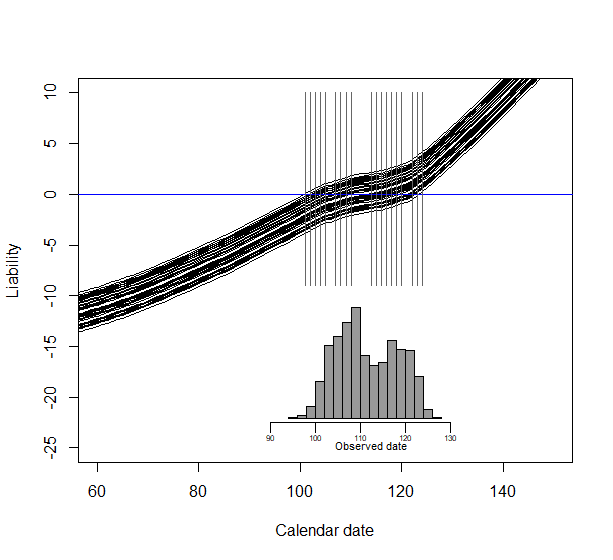


**Appendix S4: Analysing the transition to breeding as a plastic threshold trait**

To evaluate and utilise the proposition to treat breeding date as the outcome of a plastic threshold trait, previous analyses that treated breeding date as a directly expressed continuously distributed trait will need to be reformulated. This can in principle be achieved by recording individuals’ observed phenotypes on a series of dates or occasions as ‘not breeding’ or ‘breeding’ (e.g. Table S2), and utilising generalized linear mixed models to estimate parameters of underlying latent liability-scale reaction norms.

The required data structures can potentially be recovered from existing or archived datasets describing observed breeding dates. For example, individual A in Table S2 would appear in such a dataset with a breeding date of day 10, but its data could be reformulated as sequential observations of zeros (denoting ‘not breeding’) followed by ones (denoting ‘breeding’), giving 00000000001111111111 across a hypothetical 20-day survey period (Table S2).

**Table S2.** Illustrations of how breeding states, and resulting breeding dates, can be recorded and analysed. On each date or observation occasion, individuals’ states are observed as either ‘not breeding’ (0) or ‘breeding’ (1). Such observations can yield an observed breeding date (bottom row) which is typically taken as the focal trait, but can be unknown (?, i.e. unobserved) or undefined (X) if breeding never occurs. Grey shading denotes direct field observations, for example field surveys every three days. Breeding states for intervening dates can potentially be inferred from understanding of reproductive biology. Values of driving environmental variable(s) ɛ are also recorded on each date.

| Date or occasion | Individual | | | | | |  |
| --- | --- | --- | --- | --- | --- | --- | --- |
|  | A | B | C | D | E | F | ɛ |
| 1 | 0 | 0 | 0 | 0 | 1 | 0 | ɛ _1_ |
| 2 | 0 | 0 | 0 | 0 | 1 | 0 | ɛ _2_ |
| 3 | 0 | 0 | 0 | 0 | 1 | 0 | ɛ _3_ |
| 4 | 0 | 0 | 0 | 0 | 0 | 0 | ɛ _4_ |
| 5 | 0 | 0 | 0 | 0 | 0 | 0 | ɛ _5_ |
| 6 | 0 | 0 | 0 | 0 | 0 | 1 | ɛ _6_ |
| 7 | 0 | 0 | 1 | 0 | 0 | 1 | ɛ _7_ |
| 8 | 0 | 0 | 1 | 0 | 0 | 1 | ɛ _8_ |
| 9 | 0 | 0 | 1 | 0 | 0 | 1 | ɛ _9_ |
| 10 | 1 | 0 | 1 | 0 | 0 | 0 | ɛ _10_ |
| 11 | 1 | 0 | 1 | 0 | 0 | 0 | ɛ _11_ |
| 12 | 1 | 0 | 1 | 0 | 0 | 0 | ɛ _12_ |
| 13 | 1 | 0 | 1 | 0 | 0 | 0 | ɛ _13_ |
| 14 | 1 | 1 | 1 | 0 | 0 | 0 | ɛ _14_ |
| 15 | 1 | 1 | 1 | 0 | 0 | 0 | ɛ _15_ |
| 16 | 1 | 1 | 1 | 0 | 0 | 0 | ɛ _16_ |
| 17 | 1 | 1 | 1 | 0 | 0 | 0 | ɛ _17_ |
| 18 | 1 | 1 | 1 | 0 | 0 | 1 | ɛ _18_ |
| 19 | 1 | 1 | 1 | 0 | 0 | 1 | ɛ _19_ |
| 20 | 1 | 1 | 1 | 0 | 0 | 1 | ɛ _20_ |
| Breeding date | 10 | 14 | 7 | X | ? | 6 |  |

Structuring datasets in terms of observations of not breeding versus breeding rather than observed first breeding dates (e.g. Table S2) opens opportunities to include observations of individuals that would otherwise have to be excluded, or where not all information would be used. This could further reduce bias in estimates of key parameters. For example:

1) individuals that do not breed within a survey period and hence have no observable breeding date can be included with phenotypes recorded as a sequence of zeros (e.g. Table S2 individual D);

2) individuals that bred but whose breeding date was not recorded, for example because they were already breeding at the first seasonal observation and then died or failed (precluding any back-calculation of breeding date) can be recorded as ones followed by zeros (e.g. Table S2 individual E);

3) individuals that bred, ceased and bred again, can be recorded as sequences of ones then zeros then ones rather than simply recording the first breeding date (e.g. Table S2 individual F).

Further, with newly collected data or where full field records exist, it may not be necessary to explicitly record or infer first breeding date at all. Rather, individuals could simply be observed on a set of occasions (e.g. every few days, Table S2) and recorded as not breeding or breeding, and those data directly analysed. Decisions can then be taken as to whether the most powerful and least biased analyses are achieved by inferring phenotypes of not breeding or breeding on days or occasions when there was no direct observation. Such inference is very typically done in existing analyses of first breeding date where, for example, nest boxes are checked every few days and laying dates are inferred from observed numbers of eggs assuming one egg laid per day, or else inferred from subsequently observed chick hatch dates (e.g. Evans et al. 2020). Such inferences generate higher temporal resolution, but presumably also introduce error, and are not essential for a threshold trait model. Here, further temporal resolution could potentially be achieved by defining multiple thresholds representing transitions between sequential stages of reproduction (e.g. laying then incubating then hatching, or parturition then weaning). Careful initial analyses of exemplar datasets and/or simulations are now needed to establish best practice, which may depend on system-specific features including reproductive biology and the magnitude of variance in breeding date and underlying parameters.

Most simply, models aiming to estimate key quantitative genetic parameters can be formulated on the latent liability scale following Equation 1:

l_i,d_ = α_i_ + β_i_.ɛ_d_

Variance components in α and β can then be estimated from the observed phenotypes of not breeding versus breeding (i.e. zeros and ones) through a generalized linear mixed model (for example with binomial errors and a probit link function, forming a threshold trait model, de Villemereuil et al. 2016). Given observations of trajectories of the transition from not breeding to breeding across numerous relatives and non-relatives across environments, such models can be extended to separate additive genetic and permanent environmental variances in α and β (i.e. forming an ‘animal model’), and potentially even estimate GxE interactions on the latent liability scale.

Such models can be structured by a G matrix comprising additive genetic (co)variances for the liability-scale reaction norm intercept α and slope β.

σ_α_^2^ σ_α,β_

σ_α,β_ σ_β_^2^

Here, ‘date’ itself does not explicitly appear in the model or hence the analysis, but is simply used as an ordering variable to structure the dataset in terms of observation occasions (Table S2). Rather, the model explicitly includes the driving environmental variable(s) ɛ. Hence, while quantitative genetic analyses of mean breeding date interpreted as a directly expressed continuously distributed trait can be undertaken without considering any environmental variable, analyses that consider breeding date as the outcome of an environmentally-sensitive threshold trait cannot. Rather, some form of plasticity acting on the latent liability scale needs to be considered.

Here, the simplest case is that individual liability l_i_ varies linearly and directly with a single driving environmental variable ɛ. In principle, values of ɛ on each occasion could be recorded at a population level (i.e. macro-environment) or at an individual level (i.e. micro-environment, referring to particular locations). However, in practice, such driving environmental effects could be complex: potentially highly multivariate (i.e. with multiple, and potentially interacting, driving environmental variables) and/or with lagged or non-linear effects. If l_i_ is a higher order or non-linear function of ɛ, then the G-matrix would be of higher dimension. Some methods that could facilitate such analyses have already been developed and compared in animal breeding, for example utilising latent-scale splines or Legendre polynomials in longitudinal threshold models (Negussie et al. 2012; Buaban et al. 2016). These studies consider variation with time, but similar methods could be used to consider effects of driving environmental variables.

Such analyses require an appropriate start time for the trajectory towards breeding to be defined (i.e. setting the intercept α). This is important not least because the impact of variance in liability-scale reaction norm slope β on the variance in observed breeding date will depend on the timeframe over which the liability-scale reaction norm acts. In some cases, there may be a clearly biologically appropriate start time. In other cases, the form of the driving environmental variable ɛ may reduce sensitivity to the choice of start time. For example, if ɛ is taken as a measure of growing degree days (GDD), and GDD is zero until some point in early spring, then the start time before this point is effectively arbitrary.

To reveal micro-evolutionary responses to selection on the latent scale, additive genetic covariances between parameters controlling liability-scale reaction norms for the transition to breeding (i.e. α and β) and measures of fitness (w) can then be estimated through multivariate models.

Here, the G matrix would be formulated as:

σ_α_^2^ σ_α,β_ σ_α,w_

σ_α,β_ σ_β_^2^ σ_β,w_

σ_α,w_ σ_β,w_ σ_w_^2^

Additional known effects on individuals’ transitions to breeding in any year (or, conversely, on failure to breed), can be included as fixed effects or potentially as distinct traits that could covary with liability (i.e. in multivariate models). Unmeasured effects on individuals’ transitions, within or across individuals, can then be estimated as components of variance, for example associated with years or locations, and as residual variance. Models could be further extended to consider further traits that might be genetically correlated with the liability-scale reaction norm α or slope β for the transition to breeding, and/or to consider associative genetic effects of females’ mates and cross-sex genetic covariances. Such effects could generate additional latent-scale genetic constraints.

Breeding date has previously been analysed using time to event models (e.g. proportional hazards models), which also consider continuously changing environmental variables rather than specific cue periods (Gienapp et al. 2005), including non-linear effects (Gienapp et al. 2010). Such models have been used to predict future breeding dates based on climate change scenarios (Gienapp et al. 2013). However, such concepts have apparently not yet been linked to quantitative genetic analyses (in wild populations) that aim to estimate among-individual and genetic variation in reaction norm parameters underlying breeding dates.

**References**

Buaban, S., Kuchida, K., Suzuki, M., Masuda, Y., Boonkum, W. & Dunagjinda, M. (2016). Genetic analysis of the rates of conception using a longitudinal threshold model with random regression in dairy crossbreeding within a tropical environment. *Anim. Sci.* 87:961-971.

de Villemereuil, P., Schielzeth, H., Nakagawa, S. & Morrissey, M. (2016). General methods for evolutionary quantitative genetic inference from generalized mixed models. *Genetics* 204:1281-1294.

Evans, S.R., Postma, E. & Sheldon, B.C. (2020). It takes two: Heritable male effects on reproductive timing but not clutch size in a wild bird population. *Evolution* 74:2320-2331.

Gienapp, P., Hemerik, L. & Visser, M.E. (2005). A new statistical tool to predict phenology under climate change scenarios. *Glob. Change Biol.* 11:600-606.

Gienapp, P., Lof, M., Reed, T.E., McNamara, J., Verhulst, S. & Visser M.E. (2013). Predicting demographically sustainable rates of adaptation: can great tit breeding time keep pace with climate change? *Phil. Trans. R. Soc. B* 268:20120289.

Gienapp, P., Väisänen, R.A., & Brommer, J.E. (2010). Latitudinal variation in breeding time reaction norms in a passerine bird. *J. Anim. Ecol.* 79:836-842.

Negussie, E., Strandén, I., Tsuruta, S. & Mäntysaari, E.A. (2012). Longitudinal threshold model analysis of clinical mastitis using linear splines. *Livestock Sci.* 149:173-179.
